# Lack of residents due to COVID-19 pandemic. Can a mentor–mentee program during medical studies have a positive influence on the choice for specialist training in gynecology and obstetrics? A review of current literature and results of a national wide survey of medical students

**DOI:** 10.1007/s00404-021-06336-9

**Published:** 2021-12-04

**Authors:** Stefan Hertling

**Affiliations:** 1 Department of Obstetrics and Gynaecology, Jena University Hospital, Friedrich Schiller University Jena, Am Klinikum 1, 07747 Jena, Germany; 2 Department of Orthopaedics, Campus Eisenberg, University Hospital Jena, Eisenberg, Germany

**Keywords:** COVID-19 pandemic, Shortage of doctors, Students, Residents, Mentoring

## Abstract

**Objective:**

The COVID-19 pandemic restricting clinical practice and exacerbating the lack of medical staff. There is currently a lack of young residents who are deciding on further training in gynecology and obstetrics. Design: review and prospective, cross-sectional study. Setting: the aim of this study was to investigate if structured mentoring programs can counteract this deficiency. Population: medical students took part from Germany in the clinical phase.

**Methods:**

An anonymous questionnaire was developed and distributed to students from January to October 2020. Epidemiological data, questions about mentoring experiences, necessity and their expected influence on career planning were collected and statistically evaluated. Main outcome measures: structured mentoring-programs can influence the choice of subject. In particular, men are still underrepresented. Research on the topic of mentoring during in the field of gynaecology and obstetrics is completely lacking.

**Results:**

A representative number of 927 medical students took part in the survey. 22% (170/906) of the students had already participated in a mentoring program with a significantly higher proportion of men (69%; 117/170; *p* < 0.001). Of these, 94% (453/170) said this was helpful. 6% (55/906) wanted to pursue a career in gynecology and obstetrics. When asked about their appreciation for structured mentoring programs in gynecology and obstetrics, 95% (880/906) would participate and 94% agreed (871/906) that this could have an impact on their choice of specialist and career planning.

**Conclusions:**

An active provision of mentoring programs and more content can be a way of counteracting the shortage of residents in gynecology and obstetrics.

## Introduction

The COVID-19 pandemic affecting clinical practice everyday. There is a shortage of staff at every level of competence. Der There was already a shortage of staff before the corona crisis. However, the situation of the prevailing shortage of doctors was exacerbated by the SARS-COV-2 virus and shows more deficits [1]. The problem of the shortage of doctors has been the subject of controversial discussion in Germany for years and the various positions of the interest groups involved have not been resolved. The question of the current and expected future needs of doctors is of central importance because this is a basic requirement for adequate and necessary medical care for the population [2]. The Association of Senior Hospital Doctors predicted a shortage of around 17,500 doctors in the next 10 years. Many activities and campaigns, also at the federal level, were initiated to be able to win over future doctors in Germany. Some departments are less affected than others. There is a clear shortage of doctors in the fields of internal medicine, surgery, gynecology and obstetrics. Most of the vacancies for assistant doctors and specialists are currently available here [3]. The vacancies are the most difficult to fill in the field of surgery, gynecology and obstetrics [4]. Different reasons are known for this purpose: the poor conditions during training with a heavy workload with unattractive working hours and a lack of work-life balance [5]. Young medical professionals have specific ideas about their future professional prospects as doctors. The highest priority for the students is the compatibility of family and work. The desire for regular and flexible working hours is expressed below. Regardless of the expectations of the future profession as a medical professional, the tendency towards a later choice of specialization varies. The most popular training opportunities are internal medicine, pediatric and adolescent medicine and general medicine. The specialist area of gynecology and obstetrics is one of the ten most popular specialist training courses. Compared to previous years, our department is becoming less attractive during your studies [6]. The loss of attractiveness increases with the number of semesters: at the beginning of medical studies, a third of all students can imagine further training as a specialist in gynecology and obstetrics, while this initial interest is almost halved by the end of the practical year [6]. According to this, the experiences during their studies, especially during the practical year, seem to have led to a decline in the preference of students for further training in gynecology and obstetrics. Regardless of the choice of specialty, various framework conditions for satisfactory further training are considered and sometimes referred to as indispensable by future doctors. The desire for a mentor as a contact person, therefore, has the highest priority [7]. In academic medicine, several international career studies have confirmed that mentoring plays a crucial role [8, 9]. For about fifteen years now, mentoring programs for medical students have been increasingly developed in Germany. In reality, it turns out that the budding physicians subjectively receive too little support and recognition for the work they have done. In addition, many students at the beginning and during their studies lack a view of the professional perspective and an idea of future everyday work [10]. The basis of successful mentoring is the personal, continuous relationship in the professional context between mentor and mentee, which develops steadily over time. To build a trust-based relationship between mentor and mentee, certain requirements are placed on both persons. The mentor takes an active role in the professional and personal development of his protégé. He serves as a role model, motivates, strengthens and helps the mentee to develop his potential. The mentee is the focus with his questions and challenges and should accept and accept criticism. Basic elements of a mentor relationship are described in the literature [11]. The aim of this study is to use a nationwide survey of German medical students to investigate whether mentoring programs are known to date and whether these determine current career planning. In addition, the aim of this study is to check whether the students are of the opinion that specific mentoring programs from gynecology and obstetrics can change the future career planning of the students and thus change their choice of the specialist in favor of gynecology and obstetrics.

## Materials and methods

### Review

Pubmed was searched for “mentoring” and “gynaecology” and “mentoring” and “obstetrics”. 404 manuscripts were found. Among them 24 reviews, of which ten manuscripts on the fundamental subject of mentoring and again six related to the subject. The main statements of the reviews were mentioned. An overview of published overviews of mentoring programs related to the field of gynecology and obstetrics is shown in Table 1.

**Table 1:** Published reviews of mentoring programs related to the field of gynecology and obstretics in the last 35 years

Author	Year	Main message	Included sources
Royce et al. [27]	2021	The content is mentoring programs for medical students who are interested in specialist training in gynecology and obstetrics and who want to apply. The authors describe a model for faculty career counselors that is different from mentors or general academic counselors	77
Louie et al. [28]	2019	The authors describe the millennial generation and define their strengths, which can be used to improve medical and surgical education and career development	33
Bernardi et al. [29]	2019	The authors describe the situation of women in academic surgery who, despite the increasing proportion of female surgeons, are still underrepresented. Gynecology and obstetrics are counted as a sub-discipline of surgery. These are publications and leadership positions are used to recruit and promote academic surgeons. We have attempted to determine the inequality of female authorship versus male authors in peer-reviewed surgical publications	41
Hughey et al. [30]	2019	The authors describe a 2010 student program at the University of Michigan Medical School (UMMS) addressing the Global Health and Disparities (GHD) Path of Excellence as part of a broader curriculum transformation. This focuses on the relationship between the medical faculty and its students. The GHD Path is a co-curriculum with the aim of improving health disparities in the US and abroad	34
Schreuder et al. [31]	2011	The aim of the authors is to facilitate and improve the implementation of structured robotic surgical training programs. To this end, they provide an overview of established programs. The topic of mentoring is addressed. In terms of content, only mentoring programs relating to minimally invasive surgery are presented	118
Wortman et al. [32]	2010	The authors address office-based surgery (OBS) in gynecology and obstetrics. Residency programs and professional societies are encouraged to provide training in OBS surgery and develop programs to care for the next generation of physicians. Mentoring is rarely discussed here and only relates to a selected number of residents	68
Ogur et al. [33]	2007	The authors describe the integrity and benefits of the traineeship at Harvard Medical School in Cambridge (HMS-CIC) in the final year of medical school, where students learn through close and continuous contact with patients in the disciplines of internal medicine, neurology, obstetrics, gynecology, pediatrics, and psychiatry. The aim is to give the students more self-confidence in dealing with patients. This is not a classic mentoring program and only gynecology is shown	37
Gambadauro et al. [34]	2013	The authors describe the new technological developments in the field of surgical applications related to telemedicine and other surgical innovations that benefit from advances in telecommunications, and present data from a quantitative bibliographic analysis. A number of applications such as telementoring, teleproctoring and robotic telesurgery are described and their enormous potential is discussed. The aspect of mentoring is touched upon here. Contents on obstetrics are missing	66
Lefebvre et al. [35]	2016	The authors describe the use of a mentoring program to improve surgical training during the internship and show how this leads to the continuous professional development of confident gynecological surgeons	68
Fenner et al. [36]	2006	The authors deal with the topic of mentoring in gynecological surgery from the perspective of the mentor. In the operating theater, the mentor has to constantly guide, criticize and actively teach his mentee	47

### Questionnaire draft

The study questionnaire was designed in a web-based design in relation to published questionnaire research guidelines [12–14]. The choice of questions was based on both comparable work and on the quality criteria for online questionnaires [15]. The survey was created in SurveyMonkey™ (SurveyMonkey, San Mateo, CA).

### Survey conducting

The survey was distributed to all 36 medical faculties in Germany. The survey lasted from January to October 2020. Medical students in the clinical phase of their studies were included. With 36,836 medical students in the clinical section, a 95% confidence interval and an error rate of 2.5, the target number was 920. Thus, the online survey can be considered representative of the medical student population in the clinical section. The questionnaire was distributed to all registered students via e-mail mailing lists of the Student Councils. In an information letter, participants were informed that their data will be treated strictly confidential and anonymised. Access to the study was granted with a survey link and a QR code in the cover letter. The responsible local Ethics Committee was informed and did not object to the study (Reg. No.: 2019-1456-Bef.).

### Content of the study questionnaire

Based on a comprehensive list of questions based on published research findings on mentoring among medical students, a self-administered 12-point online questionnaire was developed. Members of the teaching working group of the DGGG Young Forum were invited to the validation process to provide feedback on the question format, completeness, clarity and process [16]. As a result, the questionnaire was refined. It consisted of binominal questions and questions in categoric Likert scales (5 steps) and open-ended questions and was entitled “Mentoring Programs for a Career in Gynaecology and Obstetrics.” The main sections were:

1. Respondent demographics: epidemiological data according to gender (male, female, divers).

2. Mentoring relationships: participation in a mentoring program in the past, benefits of the mentoring program, positive promotion of choice of specialization.

3. Future career choice: currently desired subject.

4. Mentoring relationship in student education: desire to participate in a structured mentoring program Gynaecology and Obstetrics, acceptance of the positive influence on the choice of the discipline Gynaecology and Obstetrics.

5. Deficit survey: questions on the reasons for the prevailing situation in the form of open questions.

The aim was to shorten the duration of the survey to a maximum of 10 min to keep the withdrawal rate as low as possible and to motivate the respondents to answer the questions as much as possible [17]. Open questions have also been implemented [18]. All participants gave consent. There were no exclusion criteria. The questionnaire was distributed via e-mail lists of the students. In an information letter, participants were informed that their data would be treated strictly confidential and anonymized. Access to the study was granted with a survey link or QR-code.

### Data analysis

Only fully completed questionnaires were included in the analysis below. The results were analyzed using SurveyMonkey TM and the Statistical Package for the Social Sciences, SPSS (Version 17. 0, SPSS Inc., Chicago, IL, USA). *p* values were calculated using the Chi^2^ test and the Levenne test. A *p* value of less than 0. 05 was considered significant.

## Results

### Review

The number of manuscripts in the last 35 years is small, although in the last 5 years there has been a significant increase in the literature on mentoring in gynecology and obstetrics. This issue seems to be a modern one. Other disciplines have focused much more on the subject, such as internal medicine, general medicine or anaesthesia and intensive care medicine. If one examines the work considered more closely, one will find content on the subject of gynecological surgery for doctors in further training. Mentoring programmes for doctors in further training in conservative gynaecology and obstetrics are presented with little or no content. Work related to a student mentoring programme is oriented towards interdisciplinary learning programmes on gynaecology and obstetrics. Work on the topic of mentoring during medical studies in the field of conservative gynaecology and obstetrics is completely lacking.

### Mentee-mentor study

Of the 98,736 medical students enrolled (2019/2020) at German medical faculties, 36,836 students were in the clinical phase of their studies. Of these, 927 students were interviewed in the clinical section. This corresponded to a percentage of 2.51%.

### Quantitative data

#### Demographic data of respondents

**Fig. 1:**
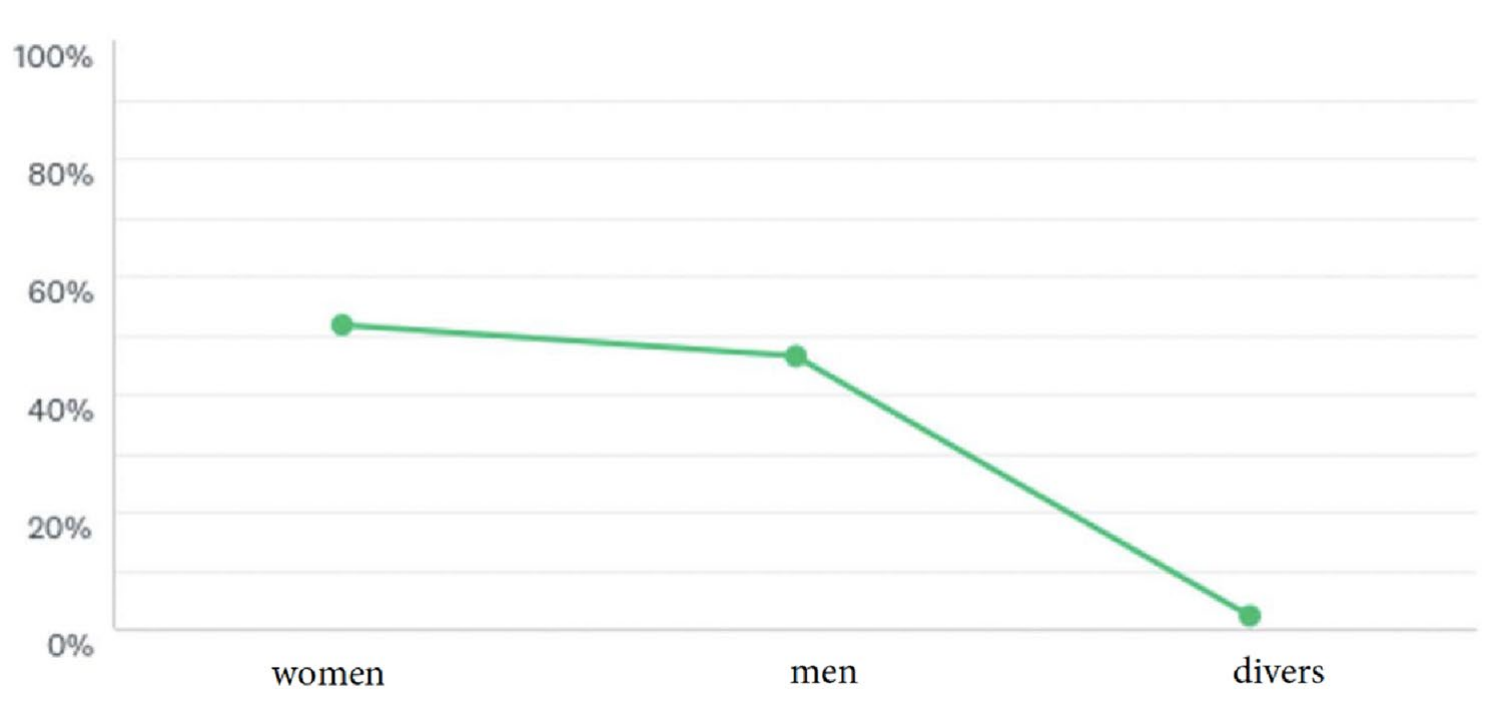
Epidemiological data 429 men (46%) and 478 women (52%) and 20 divers (2%)

The survey showed a total of 927 students of human medicine. Of these, 429 were men (46%), 499 women (52%) and 21 divers (2%) (Fig. 1). At the time of the survey, all of the students surveyed were in the clinical phase of their studies. For further statistical calculations, only the number of female and male students has been taken into account.

#### Mentoring relationships

**Fig. 2:**
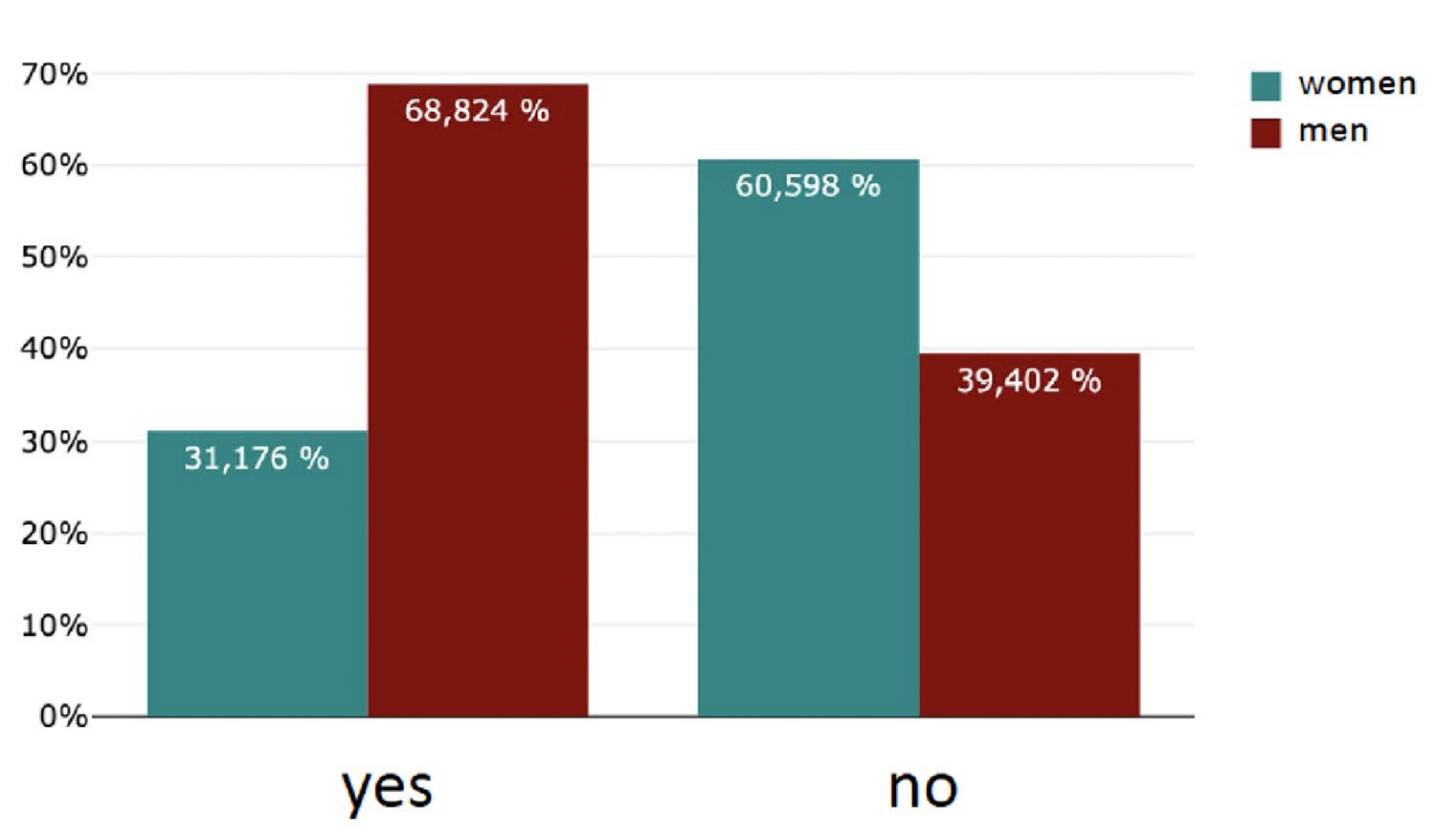
Have you ever participated in a mentoring program before?

**Fig. 3:**
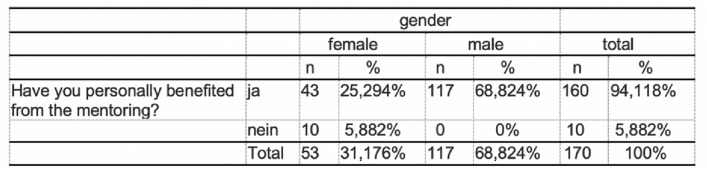
Have you personally benefited from the mentoring?

**Fig. 4:**
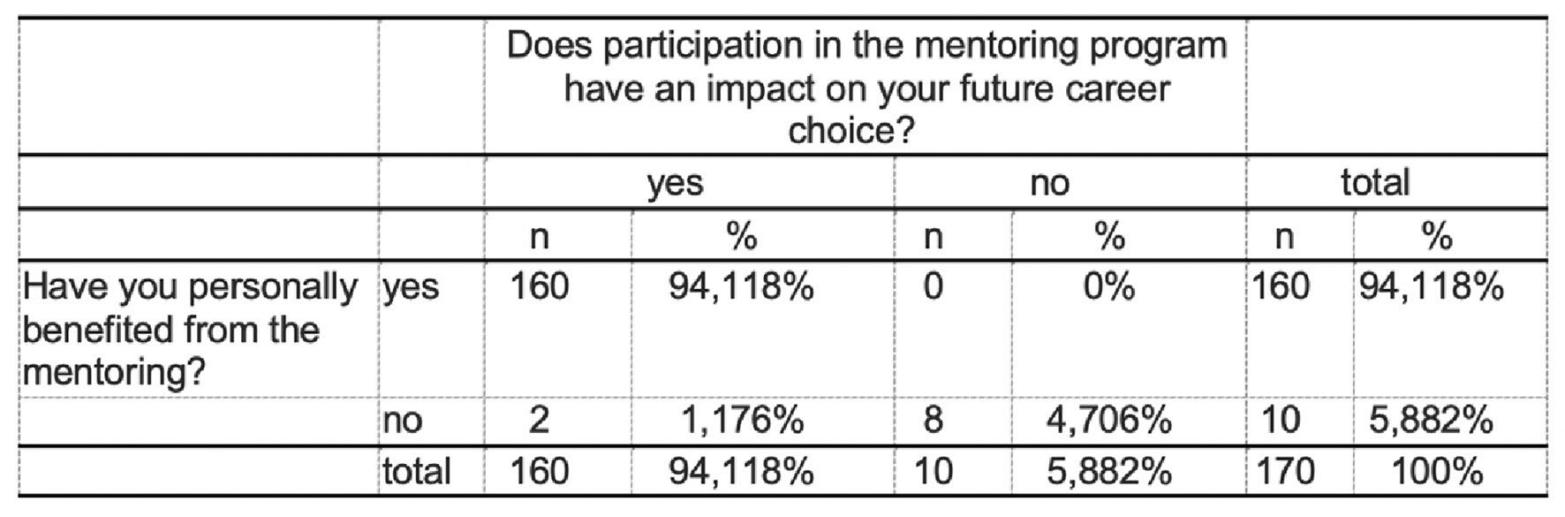
Relationship between mentoring participation and future career choice

**Fig. 5:**
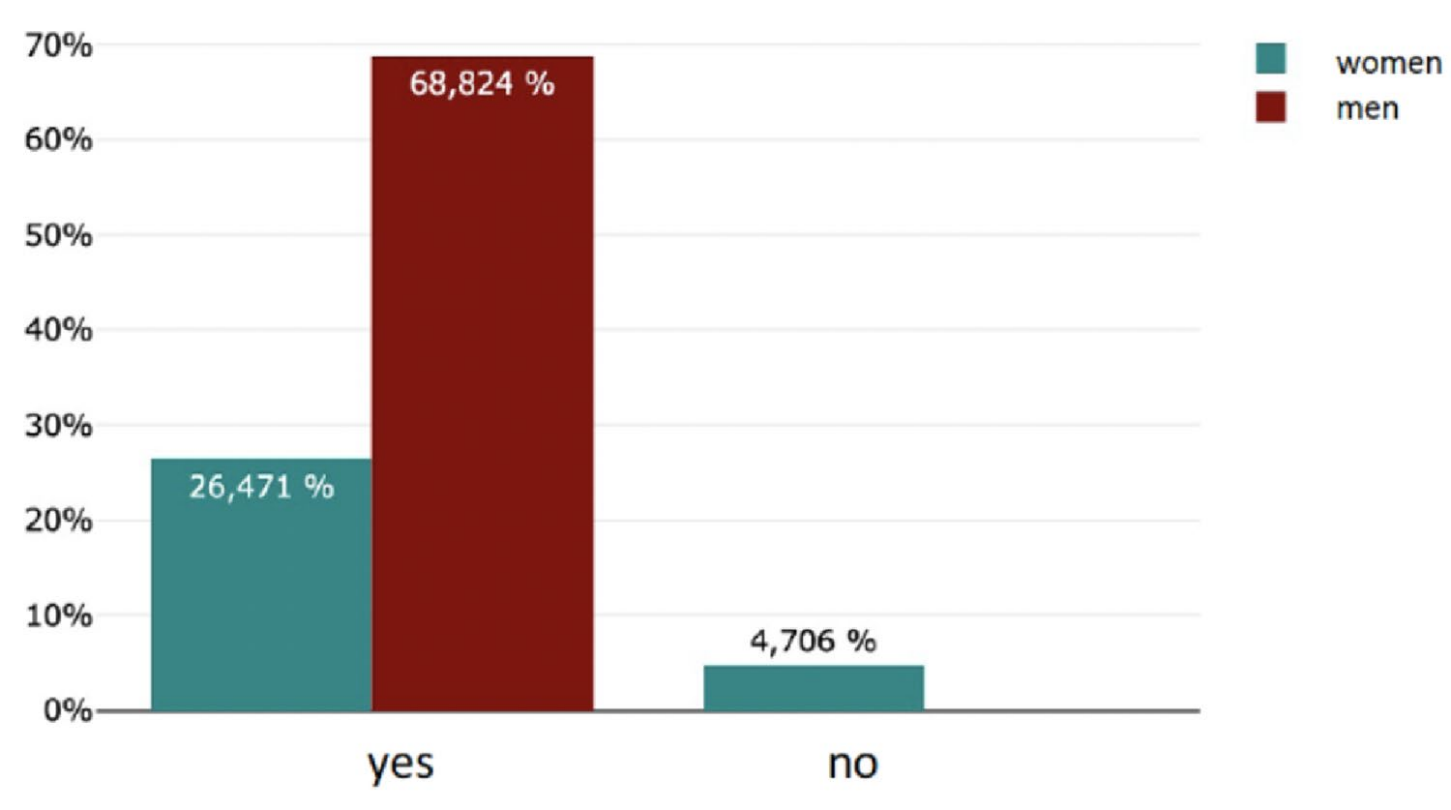
Relationship between participation in a mentoring program and career choice

The majority (78%, 736/906) of the respondents did not have a mentor–mentee relationship. On the other hand, almost 22% (170/906) have taken part in a mentoring programme (Fig. 2). Significant more male students had taken part in a mentoring program during their studies (*m* = 117, *f* = 53; *p* < 0.05). 94% see participation in a mentoring programme (160/170) as helpful with personal benefit and 94% (160/170) confirm that the mentoring relationship has had an impact on the subsequent choice of career specialisation (Fig. 3). There was a significant difference in gender (*f* = 53, *m* = 117; *p* < 0.001). Only 4. 7% (33/738) saw no link between participation in a mentoring programme and their subsequent professional specialisation (Figs. 4, 5).

#### Gynaecology and obstetrics

**Fig. 6:**
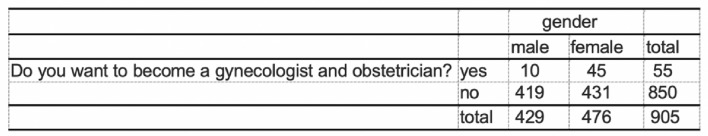
Later choice of profession

**Fig. 7:**
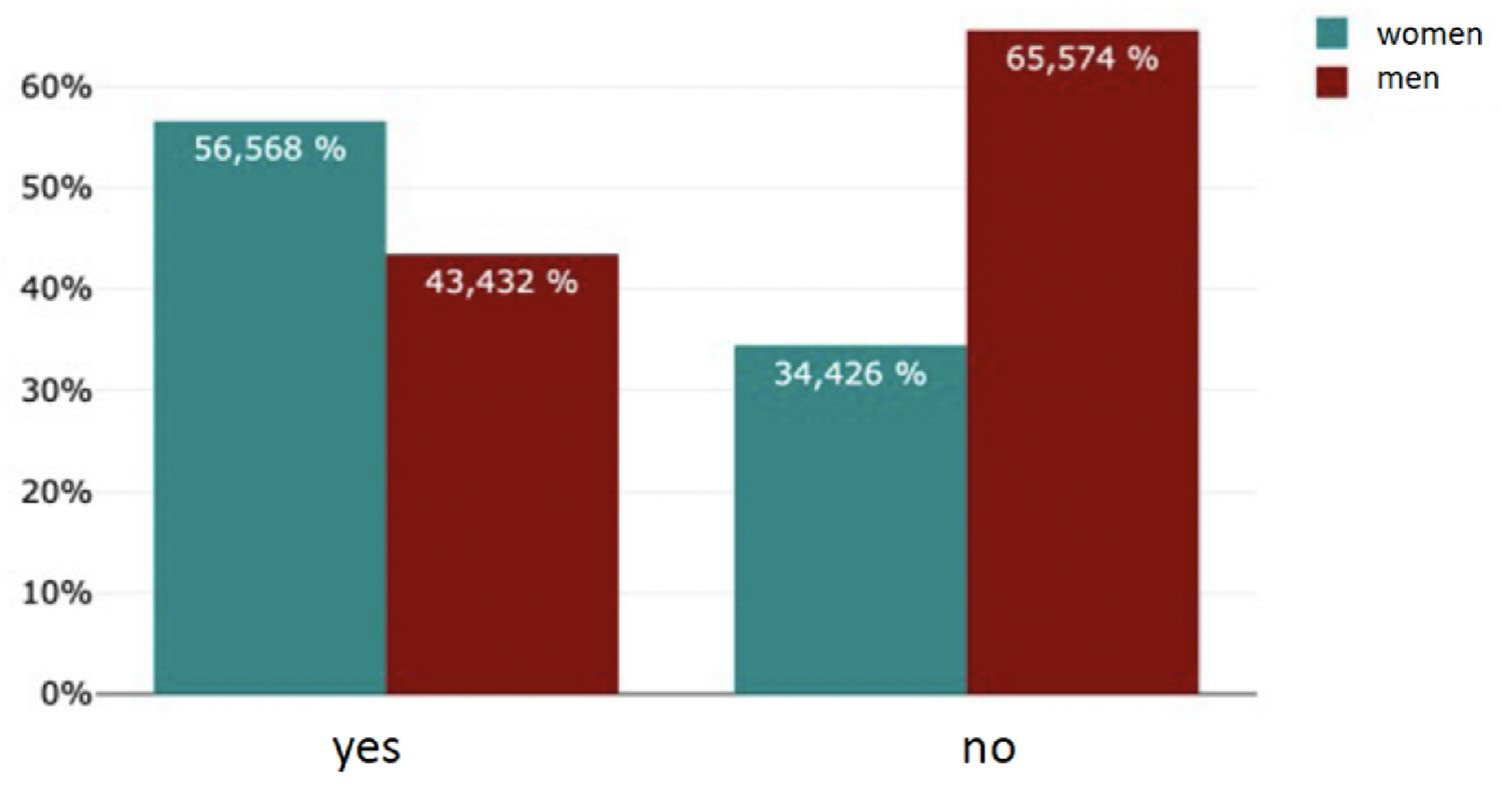
Interest in structured mentoring programs in gynaecology and obstetrics

**Fig. 8:**
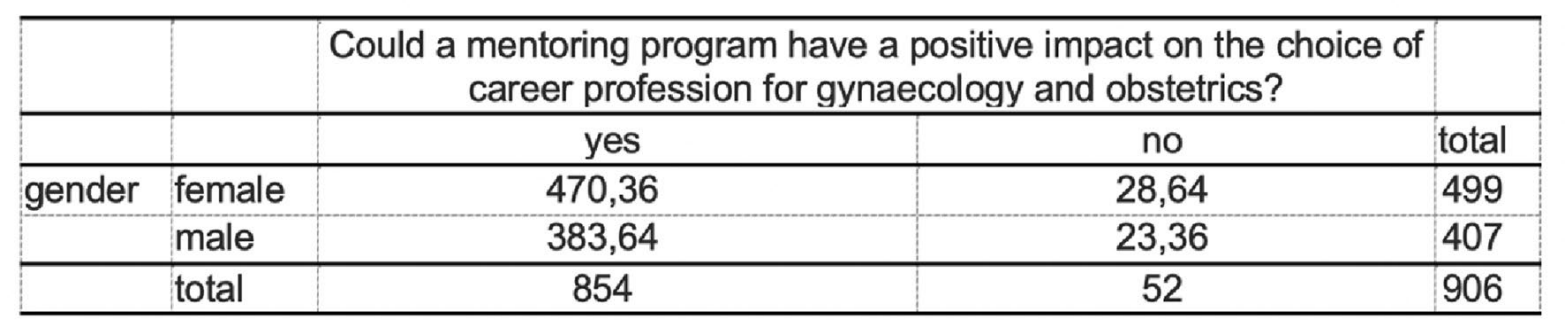
Could have a posivitve impact on their choice of specialist and career planning in the department of gynaecology and obstetrics

**Fig. 9:**
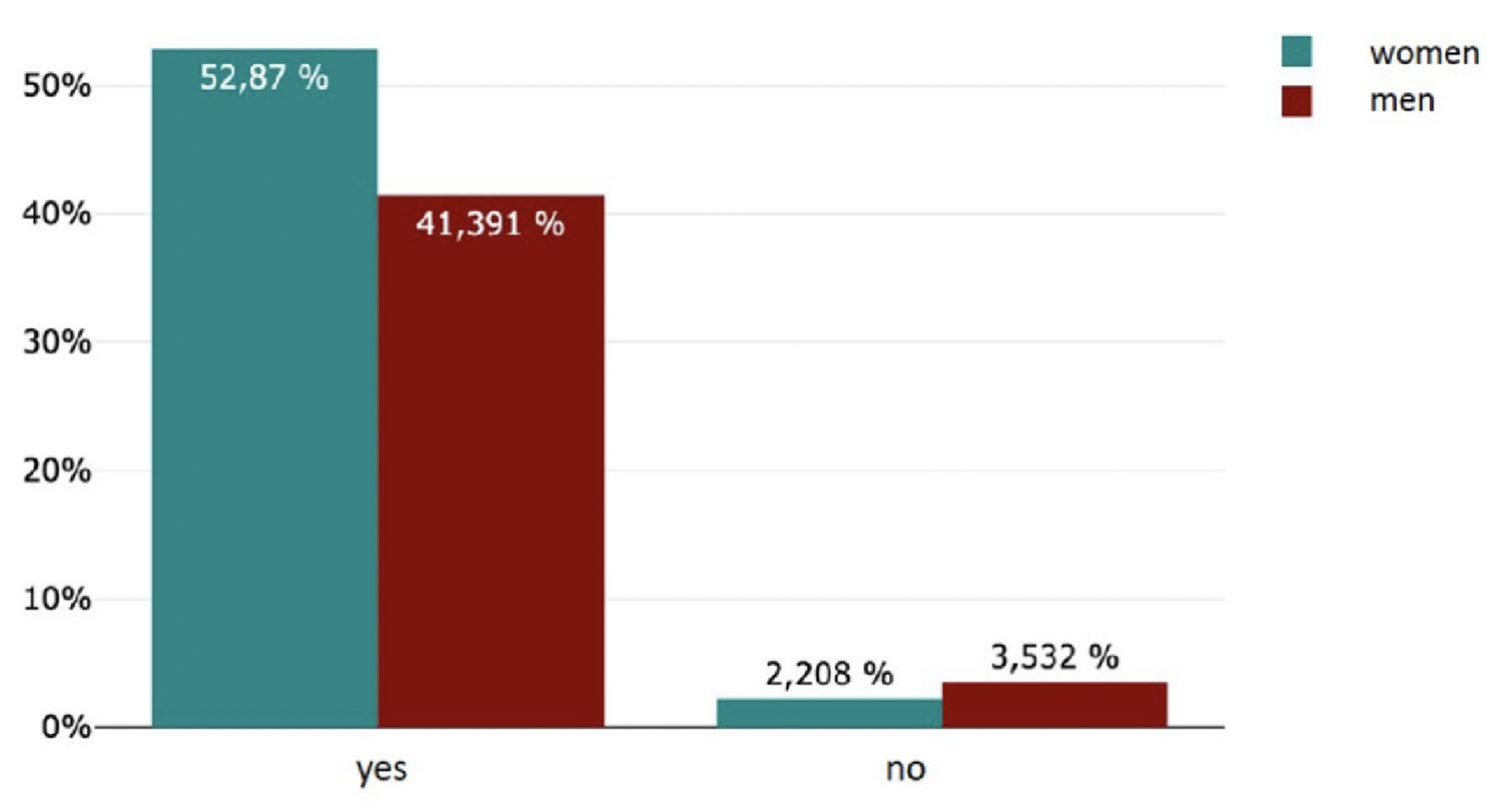
Positive impact on choice of specialist and career planning in the department of gynaecology and obstetrics

Six percent (55/905) of the respondents want to pursue a career in gynecology and obstetrics. Of these 55 respondents, 82% were women (45/55) and 18% were men (10/55) (Fig. 6). Overall, 4.97 percent of the female students surveyed and 1.10 percent of the male students surveyed aspire to a specialist in gynaecology and obstetrics. There was a significant difference in gender (*f* = 45, *m* = 10; *p* < 0.001). When asked about their interest in structured mentoring programs in gynaecology and obstetrics, almost 95% of all respondents (858/906) would participate. There was a significant difference in gender (*f* = 478, *m* = 376; *p* < 0.001) (Fig. 7). Almost 94% agreed (854/906) that participation in a structured mentoring program in the department of gynecology and obstetrics could have a positive impact on their choice of specialist and career planning in the department of gynecology and obstetrics. There was a significant difference in sex (*m* = 375, *f* = 479; *p* < 0.013) (Figs. 8, 9).

### Qualitative data

Open questions were asked about the reasons for the lack of interest in specialist training in gynaecology and obstetrics and about suggestions for improvement from a student perspective. When asked about possible reasons from the students' point of view why few students choose gynaecology and obstetrics, the students' main criticism came from the general high workload, unplanned working hours and the subsequent difficult reconciliation of work and family.

“Gynaecology is a very interesting field but, as in all surgical subjects, the high workload characterises the clinical work routine”.

“All medical disciplines have a high number of patient pass-throughs, but in obstetrics the working hours and workload are unpredictable, children come when they want”.

“From experience in my own family, I see how hard it can be to combine private and professional life. I don’t want this later”.

When asked what they wanted for a mentoring program in gynaecology and obstetrics, the majority of students agreed that they felt a high level of practical relevance coupled with a mentor who felt responsible for them, with whom they could build a personal relationship without feeling underscored.

“The ideal idea of a mentoring program in gynaecology and obstetrics is practice-oriented teaching in theory and at the patient’s bedside. We should discuss real patient cases here”.

“The personal level of the mentor strongly influences a mentor program. If it is professional but appreciative, the department plays a subordinate role”.

From a student’s point of view, there are many suggestions to improve the lack of interest in training in gynaecology and obstetrics. For many students the faculty is clear, but they have few points of contact during their studies. Circular internships as well as other practical gynaecological teaching contents are not available. Therefore, more practical content should be offered, even beyond the university level.

“Gynaecology is a surgical discipline. However, I didn’t see much of this during my studies. Either the internship was cancelled or we discussed cases on paper. So the two weeks of internship went by somehow”.

In contact with gynaecologists and obstetricians, there is a lack of role models. Although there are many female interns in the clinics, the picture is different on the executive levels. There is a lack of female pioneers for female students.

“Are there only male chief doctors? I thought it was different in gynecology and obstetrics. We have had many female doctors in training. Where are the female chief physicians and senior physicians? You can act as role models for us female students”.

Male students often reported being sent out during examinations or births. Practical teaching content could not be taken away and created an unpleasant situation. A general gender-neutral view would be one option.

“There are great debates about gendering these days. Whole newspapers are full of it. But as a male student, I’m being sent out during a medical check-up as part of my student internship or when I’m having a child, with the argument that I’m a man. But how am I supposed to be able to find a specialty by actively sending out? General and absolute equality would be the first step towards improvement”.

“A personal mentor would be a great opportunity to look outside the box and get to know the field in peace. But this requires willing doctors who are available to do something like this despite the daily clinical routine and not half-hearted and annoyed”.

## Discussion

In a multi-center survey of all students who were enrolled in medical faculties in the clinical section of Germany in the winter semester 2019/2020 and summer semester 2020, this study was intended to provide an overview of the participation of medical students in mentoring programs and their impact on the planning of their specialist training. Mentoring programmes could influence the choice of specialisation in the field [19]. Knowledge of the impact of mentoring programmes on basic medical education could be used to guide young people even in disciplines with a shortage of young talent. As far as the authors are aware, this is the first representative study that deals with the current prevalence of mentors among medical students and at the same time includes the choice of specialization for a professional career in the field of gynecology and obstetrics. In the Western world, high workload and low work-life balance are described as the main reasons why medical students do not plan a career in gynecology and obstetrics [20]. Analysis of the online survey by gender reveals an under-representation of women (52%) in the study. As women are significantly more represented among medical students, with up to 80%, an even distribution of women is still under-represented. In the past, women, in particular, showed significantly lower participation in mentoring programs in gynaecology and obstetrics, despite the great interest in structured mentoring programs in the field of gynaecology and obstetrics when they were able to make a decision. The study also showed that women are more likely than men to pursue a career in gynecology and obstetrics and that they are much more likely to opt for this discipline. The proportion of men who seek specialist training in gynaecology and obstetrics in the course of their studies has been consistently low for years [21]. In the literature, men are still a minority in gynecology and obstetrics, accounting for 14% of gynecologists in the USA and 16% in Germany [22]. The reasons for this are manifold: lack of basic interest, awareness of the challenges of a lifestyle within specialist training to become gynaecologists and obstetricians and a lack of male role models and mentoring in the literature [21, 22]. In individual cases, men have deliberately decided against choosing a specialist to become a gynaecologist and obstetrician on the grounds of gender discrimination or fear of later legal consequences due to the male sex in clinical practice in the treatment of women [23]. Interesting, however, was the large number of students who were interested in the field of gynecology and obstetrics. There was no evidence of a decline in interest during the course of the studies reported in the literature [24]. Looking at the period of study, it can be seen that higher semesters attach great importance to mentoring programs. Mentoring programs are important in all periods of study, but seem to become even more important with the later semesters. Some studies have assumed that students decide on their field of study at the end of their studies or after completing their medical studies, others have indicated that they make decisions about their future medical careers during or even before their medical studies. Other studies have shown that the majority of students have not yet chosen a subject in the past year [25]. Many factors influence the choice after specialist training, e. g. support for doctoral theses or preparation for examinations. Since the choice of profession within the scope of medical studies is also made individually, a mentoring programme should also be individually accessible in each study period [26]. The mentoring relationships were not recorded in more detail and qualitative (type of mentoring relationship, mentoring goals) or quantitative (frequency of meetings, level of hierarchy of the mentor-mentee relationship) criteria of the mentoring relationship were not queried. However, the simplification of the questionnaire meant that it was not possible to ask about the modalities of mentoring programmes. Therefore, this study is unable to assess the availability, structure and design of mentoring programs. Finally, mentoring programs can have a positive impact on career planning. To become more active in this direction, to offer mentoring programmes and to offer more gynaecological and obstetric content, can be a way to counteract the shortage of trainees. Men at medical schools generally participate more often in mentoring programmes, but only in a few cases do they seek specialist training to become gynaecologists and obstetricians. Therefore, men are a suitable group for mentoring offers to attract them to postgraduate gynaecological and obstetric education. Students in clinical semesters are generally interested in participating in mentoring programs from the specialist field of gynecology and obstetrics so that this creates a basis for offering structured mentoring programs and especially for undecided medical students due to the influence of mentoring programs from the gynaecological and obstetrics specialist to be able to inspire.

## Conclusion

Due to the current COVID-19 pandemic, the shortage of doctors is worsening. Not all clinics or practices per se are affected by this. Nevertheless, the issue of the lack of medical staff remains omnipresent and will become even more apparent in the coming years than it has already been. At the federal level, this issue has been discussed several times and different approaches have been sought. Practical and solid solutions seem far away. The Department of Gynaecology and Obstetrics is also affected. For example, vacancies for interns are more difficult to fill than vacancies in most other medical fields. Structured mentoring programs from the Department of Gynaecology and Obstetrics for students could be a solution. Student interest in mentoring programs is present and can be an important step in the work against the shortage of doctors. These structured student programmes could be organised together with the professional societies and the medical faculties and implemented during the student’s education. The field of gynecology and obstetrics has the highest female quota of more than 80%. Four out of five doctors who take their exams in gynaecology and obstetrics are women. Only one out of five doctors are aspiring gynaecologists and obstetricians. Only just under two percent of male students aspire to a specialist training in gynaecology and obstetrics in the clinical part of their studies. Few male students are interested in specialist training and a small number of male. Specific structural mentoring programmes for male students may be offered to stimulate interest in the field of gynaecology and obstetrics, to create possible missing role models and to eliminate outdated ways of thinking related to prejudices or gender in the field of gynaecology and obstetrics. Obstetrics. Other medical faculties actively address the staffing situation and offer structured mentoring programs for interested students at medical faculties and benefit from them. To become more active in this direction, to offer mentoring programmes and to offer more gynaecological and obstetric content, can be a way to counteract the shortage of trainees. Men in medical schools were more likely to participate in mentoring programmes, so men are a suitable group for mentoring opportunities to attract them to postgraduate education in gynaecology and obstetrics.

## Data Availability

All data relevant to the study are included in the article or uploaded as supplementary information. Data are available on reasonable request. The data will be shared if there is a reasonable request for it.

## Declarations

### Provenance and peer review

Not commissioned; externally peer reviewed.
